# The Synergistic Enhancing-Memory Effect of Donepezil and S 38093 (a Histamine H_3_ Antagonist) Is Mediated by Increased Neural Activity in the Septo-hippocampal Circuitry in Middle-Aged Mice

**DOI:** 10.3389/fphar.2016.00492

**Published:** 2016-12-22

**Authors:** Aurore Sors, Ali Krazem, Jan Kehr, Takashi Yoshitake, Gaelle Dominguez, Nadia Henkous, Claire Letondor, Elisabeth Mocaer, Daniel J. Béracochéa

**Affiliations:** ^1^Pôle d’Innovation Thérapeutique Neuropsychiatrie ServierSuresnes, France; ^2^CNRS 5287, Institut de Neurosciences Cognitives et Intégratives d’Aquitaine, Université de BordeauxUMR, Pessac, France; ^3^Pronexus Analytical ABBromma, Sweden; ^4^Section of Pharmacological Neurochemistry, Department of Physiology and Pharmacology, Karolinska InstituteStockholm, Sweden

**Keywords:** cognitive aging, amnesia, acetylcholine, histamine, microdialysis, CREB, hippocampus, septum

## Abstract

Donepezil, an acetylcholinesterase inhibitor, induces only moderate symptomatic effects on memory in Alzheimer’s disease patients. An alternative strategy for treatment of cognitive symptoms could be to act simultaneously on both histaminergic and cholinergic pathways, to create a synergistic effect. To that aim, 14 month old C57/Bl6 mice were administered per oesophagy during nine consecutive days with Donepezil (at 0.1 and 0.3 mg/kg) and S 38093 (at 0.1, 0.3, and 1.0 mg/kg), a H3 histaminergic antagonist developed by Servier, alone or in combination and tested for memory in a contextual memory task that modelized the age-induced memory dysfunction. The present study shows that the combination of Donepezil and S 38093 induced a dose-dependent synergistic memory-enhancing effect in middle-aged mice with a statistically higher size of effect never obtained with compounds alone and without any pharmacokinetic interaction between both compounds. We demonstrated that the memory-enhancing effect of the S 38093 and Donepezil combination is mediated by its action on the septo-hippocampal circuitry, since it canceled out the reduction of CREB phosphorylation (pCREB) observed in these brain areas in vehicle-treated middle-aged animals. Overall, the effects of drug combinations on pCREB in the hippocampus indicate that the synergistic promnesiant effects of the combination on memory performance in middle-aged mice stem primarily from an enhancement of neural activity in the septo-hippocampal system.

## Introduction

The “cholinergic hypothesis” in aging or Alzheimer’s disease is based on the correlation between the memory impairment and the decrease of the cholinergic function in the brain (Bartus et al., 1982; Johannsson et al., 2015). Such correlation has also been observed in aged rodents (Fu et al., 2014; Lim et al., 2015). The first clinical approach has thus consisted in inhibiting the decrease of acetylcholine (ACh) by blocking its degradation by acetylcholinesterase (AChE) in the synaptic cleft (Marighetto et al., 2008). Acetylcholinesterase inhibitors (AChEI), such as Donepezil, are currently used in this way but are however modestly effective in AD with only moderate cognitive improvements (Lockhart and Lestage, 2003; Francis et al., 2010).

In addition to deficits in ACh, histamine neurotransmission is also reportedly diminished in elderly or in AD (Fernández-Novoa and Cacabelos, 2001). Histamine has raised interest for its implication in memory and attention (Witkin and Nelson, 2004; Schwartz, 2011). Among the four types of histaminergic receptors, the H3 subtype, mainly presynaptic, is expressed on neurons in the central nervous system (CNS), particularly in brain areas involved in cognitive processes and arousal. Expressed on histaminergic neurons, its activation leads to the inhibition of the synthesis and release of histamine (Arrang et al., 1983) and also negatively regulates the release of other neurotransmitters such as ACh when H3 is expressed on heterologous nerve endings (Blandina et al., 1996; Brown et al., 2001). Thus, it has been argued that H3 antagonists, which could hamper the constitutive negative feedback of H3 receptors on the release of these neurotransmitters, would be valuable in correcting cognitive deficiencies (Fox et al., 2003; Ligneau et al., 2007; Femenía et al., 2015).

To that aim, S 38093 was developed by Servier. S 38093 is an inverse agonist/antagonist of H3 receptors, which has shown procognitive properties at a mean pharmacological dose of 0.3 mg/kg (Panayi et al., 2014). Indeed, it improves performance in episodic-like memory paradigm both in adult rats (object recognition with natural forgetting or scopolamine-induced amnesia) and aged mice (relational memory task). It is also effective in working memory paradigms in middle-aged mice (spontaneous alternation or concurrent serial alternation) and in aged monkeys (delayed matching to sample task). These effects are thought to be mediated by an enhanced release of neurotransmitters especially ACh and histamine, which are indeed observed by microdialysis in the prefrontal cortex (PFC) and the hippocampus of rats after S 38093 administration (Panayi et al., 2014)

An interesting alternative for treatment of cognitive decline could be to act simultaneously on both histaminergic and cholinergic pathways, to create a synergistic effect. Indeed, combined treatments can be more effective than compounds alone and can allow using lower doses of each compound, i.e., minimizing the potential negative side effects. Therefore, the aim of the present study was to investigate in a first experiment the effect of the chronic administration of S 38093 and Donepezil, alone or in combination, in a model of contextual memory impairments in middle-aged mice (Tronche et al., 2010). In a second experiment, we measured the cAMP response element binding protein (CREB) phosphorylation as a marker of intracellular PKA activation and increased neuronal activity after behavioral testing. Indeed, it has been shown that memory consolidation relies on PKA activation and subsequent CREB phosphorylation in the hippocampus (Bernabeu et al., 1997; Colombo et al., 2003; Baudonnat et al., 2011) whereas cognitive abilities involving mPFC are impaired by PKA activation (Runyan and Dash, 2005; Barsegyan et al., 2010). Therefore, CREB appears as a point of convergence for the intraneuronal kinase/phosphatase balance, and reflects neuronal activity sustaining memory processes (Benito and Barco, 2010).

## Materials and Methods

### Animals

Animals were 12 months-old mice of the C57/Bl6 inbred strain obtained from Charles River (L’Arbresle, France). They were housed in collective cage in the colony room (12 h light–dark cycle) until they were 14 months. Three weeks before the experiments, they were housed individually. All procedures were carried out during the light phase of the cycle, between 08:00 a.m. and 12:00 a.m. Three days before the acquisition phase of memory testing and during the remaining behavioral phase, all subjects were maintained at 85–90% of their *ad libitum* body weight. All experiments were performed in accordance with the local Ethics Committee for Animal Experiments and the European Communities Council Directive of 1st February 2013 (2010/63/UE).

### Memory Test

The memory task and apparatus has been fully described previously (Chauveau et al., 2009). The contextual serial discrimination (CSD) task is based on two successive discriminations in a four-hole board which can be retrieved with the help of specific temporal and contextual cues associated with each of them. We already showed that unlike young mice, middle-aged mice showed a deficit in this task (Béracochéa et al., 2007, 2008, 2011; Tronche et al., 2010).

#### Acquisition Phase

**FIGURE 1:**
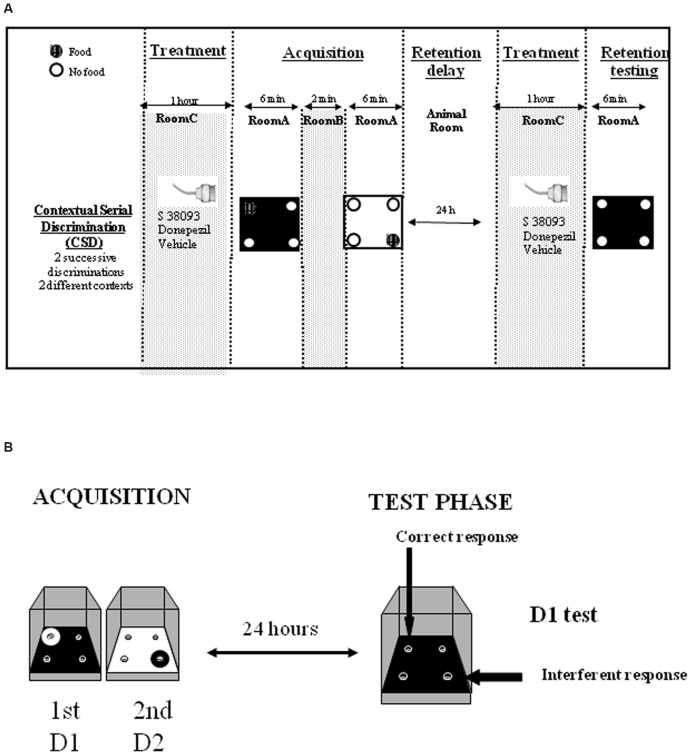
**(A)** Contextual serial discrimination: at the acquisition phase, mice performed two consecutive spatial discriminations varying by the color and texture of the floor, i.e., D1: Discrimination 1 and D2: Discrimination 2. For each discrimination, only one hole out of the four holes of the apparatus was baited (hashed circles). The two discrimination were separated by a 2 min delay interval during which animals are placed in room B. A 24-h delay was interpolated between the acquisition and test phases, during which mice were returned in the colony room. 1 h prior to acquisition and test phases, mice received a *per se* administration of the compounds or vehicle solution in a chamber placed in a room (room C) different from the one in which the behavioral experiments was conducted (room A). Subsequently, mice were submitted to the test phase in which they were replaced on the floor of the first discrimination without any food pellet in the apparatus. **(B)** Two types of responses were calculated: (i) correct responses corresponding to head-dips into the hole baited at the acquisition of the first discrimination (D1), on the same floor context; (ii) interference responses corresponding to head-dips into the hole baited at the other (second) discrimination (D2). These two parameters allow calculus of the SCM score.

The acquisition phase took place in room A where animals learned two consecutive spatial discriminations (D1 and D2; **Figure 1A**) which differed by the color and texture of the floor and were separated by a 2-min delay interval. For both D1 and D2, 10 20-mg food pellets were available during the 6-min exploration sessions; for D2 specifically, the baited hole was consistently located in the opposite symmetrical hole. Environmental cues made of colored paper sheets were positioned at 1.00 m above the board. At the end of the acquisition phase, mice returned in the animal’s room for 24 h. Animals retained for the test phase in the present study for both Experiments 1 and 2 have eaten at least 7–8 pellets/10 during both acquisitions.

#### Test Phase

Mice were replaced on the D1 floor in the board without any pellet in the apparatus and were allowed to freely explore for 6 min during which the number of head-dips in each hole was counted. This allowed measures of the % of “correct responses” (head-dips into the hole previously baited on the same floor-context), the % of “interference responses” (head-dips into the hole previously baited at D2, on the other floor-context) (**Figure 1B**) and of the “strength” of “contextual memory” score (SCM) (% correct responses - % interference responses). Thus, since correct responses are based on the use of the internal context (color of the floor) and interferent responses are based on the use of spatial allocentric cues previously associated with the other floor, thus the trend of SCM score toward a positive difference represents the gain of internal contextual memory, at the expense of allocentric spatial one.

### Experiment 1: Effect of the Compounds and Their Combinations on Age-Related Memory Deficit

#### Drug Administration

**FIGURE 2:**
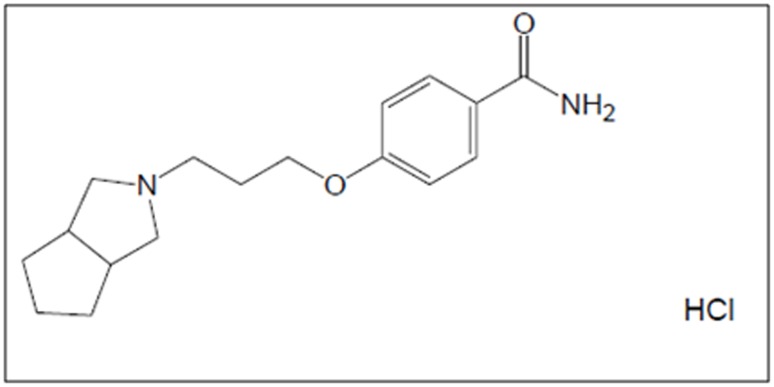
**Chemical formula of S 38093 (C_17_H_24_N_2_O_2_, HCl)**.

S 38093 (Servier, France) and Donepezil (Syntheval, France) are hydrochloride salts. S 38093 belongs to the chemical family of benzamides, its hydrochloride salt S 38093-2 (called S 38093 in the paper) was used in this study. Its chemical formula is C_17_H_24_N_2_O_2_, HCl (**Figure 2**).

S 38093 and Donepezil were diluted in purified water. Mice were allocated to administration of vehicle (purified water), S 38093 (*S1*: 0.1 mg/kg; *S2*: 0.3 mg/kg; *S3*: 1.0 mg/kg) or Donepezil (*Don1*: 0.1 mg/kg; *Don2*: 0.3 mg/kg), or a combination of both S 38093 and Donepezil at the same doses with *N* = 12 in each group. S 38093 and Donepezil doses were determined according to previous data (Béracochéa et al., 2008; Panayi et al., 2014). Before behavioral testing, all mice received for nine consecutive days a daily esophageal administration of S 38093, Donepezil, vehicle or S 38093 + Donepezil combinations, administered at a volume of 10 mL/kg. The two last administrations were delivered 1 h before the acquisition and test phases.

#### Pharmacokinetics Study

For the pharmacokinetics study, an additional day of treatment (day 10) was done for blood sampling to measure concentrations of S 38093 and Donepezil in plasma, on the combinations groups where either the most important synergistic effect was observed, or containing the highest doses of S 38093 and Donepezil. Then, blood sampling (250 μL /sample) was performed on five groups (*N* = 3 animals per group): S 38093 (0.1 and 1.0 mg/kg); Donepezil (0.3 mg/kg); combination of Donepezil (0.3 mg/kg); and S 38093 (0.1 and 1.0 mg/kg). The sampling times are 30 min, 1, 2, 4, and 6 h. In each mouse, two time points of blood sampling were performed. Plasma was extracted from the blood sample by centrifugation (+4°C, 3000 *g*, 10 min) and stored at -80°C. Then, plasma samples were sent in dry ice to MDS (MDS Pharma Services, Switzerland) for the pharmacokinetic analysis.

S 38093 and Donepezil were independently measured by liquid chromatography using tandem mass spectrometry detection (LC/MS–MS). Prior to analysis, S 38093 was extracted from 25 μL of sample by solid phase extraction on an Isolute 96 CBA SPE 50 mg cartridge while Donepezil was extracted by liquid/liquid extraction from a second 25 μl aliquot. The limit of quantification was 0.3 ng/mL for S 38093 and 0.1 ng/mL for Donepezil.

Animals were sacrificed by cervical elongation immediately after either behavioral testing or plasma sampling.

### Experiment 2: Study of the Effects of the Compounds on Age-Related Memory Deficit and CREB Phosphorylation

#### Behavioral Testing

Food deprivation, drug administration and behavioral testing procedures were similar as in Experiment1. We performed in Experiment 2 an immunohistochemical study on the combination groups where either the most important synergistic effect was observed (Don2+S1 and Don2+S2), or Vehicle, S 38093 and Donepezil alone (Vehicle, S1, S2, and Don2). Independent groups of mice (*N* = 10 per group) were used. An additional group composed by young vehicle mice (4–5 months young-vehicles, *N* = 10) was added for determination of the aging effect on both memory and phosphorylated CREB immunoreactivities, as compared to middle-aged Vehicles. For the immunohistochemical study, animals submitted to D1 memory testing were compared to “naïve” mice isolated in the colony room (with *N* = 5 per group; Naïve condition) and that underwent the food deprivation procedure and drug treatments as behaving animals (Test condition).

#### Immunohistochemistry

Thirty minutes after completion of the test session, mice were deeply anesthetized (Avertin, 10 mL/kg intraperitoneally i.p.), and perfused transcardially with an ice-cold solution of 4% paraformaldehyde in phosphate buffer (0.1 M, pH 7.4). After perfusion, brains were removed and post-fixed overnight in the same fixative at 4°C. Brains were then put in a sucrose solution (30% in Tris Buffer 0.1 M, pH 7.4) during 24 h. They were then frozen and cut in 50-mm coronal free-floating sections with a freezing microtome (Leica) to proceed to immunochemistry.

Total CREB (tCREB) and phosphorylated CREB (pCREB) immunostainings were performed as described in full previously (Vandesquille et al., 2013; Dominguez et al., 2014, 2016). Countings were made in the following brain regions according to Paxinos and Franklin (2001) atlas: the CA1 of the dorsal (dCA1) and ventral (vCA1) hippocampus, the prelimbic cortex (PL), the dorsal striatum (St), the basolateral amygdala nucleus (BLA) and the medial septum nucleus (MS). Digital images were captured at 10X magnification using an Olympus (BX50) and an imaging analysis system (ImageJ^®^). At least six serial sections for each brain region were analyzed using a computerized image analysis system (Visiolab 2000^®^, Biocom, and V4.50). Quantification was expressed as mean number of positive nuclei per mm^2^.

### Statistical Analysis

Behavioral data were analyzed by one-way or two-way factorial analyses of variance, followed when adequate, by *post hoc* (Dunnett test) comparisons using the least significant difference test, with a *p* < 0.05 statistical threshold. Data were represented as mean ± standard-error of the mean. For correlation analyses, the Spearman’s correlation coefficient, R, was determined. For immunohistochemistry, in so far as no difference was found in the naïve condition, data were analyzed similarly using the rough number of immunopositive cells, with a *p* < 0.05 statistical threshold.

## Results

### EXPERIMENT 1

#### Effect of the Compounds and Their Combinations on Age-Related Memory Deficit

Body weights among the groups were ranged from 28.9 ± 3.5 to 32.4 ± 4.3 g and no significant between-groups difference was observed [*F*(11,132) < 1.0]. During the food restriction period, both vehicles and drug-treated mice eaten all their allocated daily amount of dry food. All animals retained for the test session have eaten at least 7/8 pellets out of 10 at both the first and second acquisitions.

##### Acquisition phase

No significant difference was observed among the groups on the total number of head-dips as well as on the % exploration of the baited hole both at acquisitions 1 and 2 (*p* > 0.10 in all analyses).

##### Test phase

*Total number of head-dips*. The total number of head-dips ranged from 31.2 ± 9.4 to 62.3 ± 9.4 and no significant between-groups difference was observed [*F*(11,132) < 1.0]. The interaction (S 38093 X Donepezil) was also not significant [*F*(6,132) = 0.54, *p* = 0.780]. Thus, the dose effect of S 38093 or Donepezil has been analyzed at all levels pooled of the other drug. There was no significant effect of either S 38093 [*F*(3,132) = 1.45, *p* = 0.231] or Donepezil [*F*(2,132) = 0.28, *p* = 0.760] on this parameter.

**FIGURE 3:**
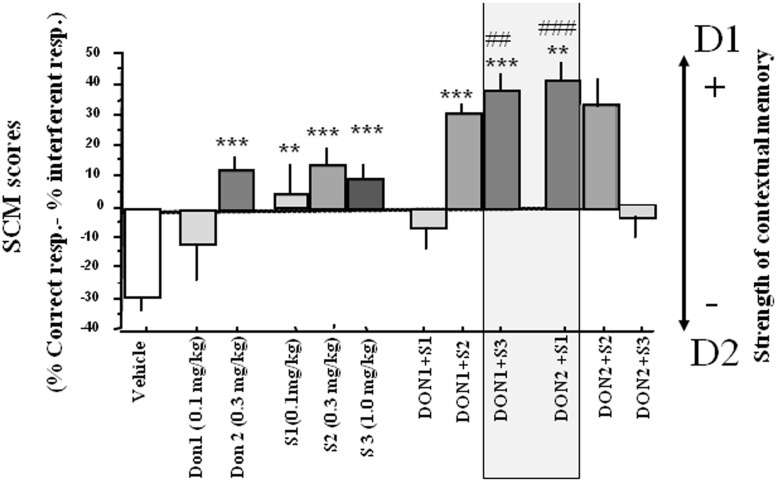
**Effects of S 38093, Donepezil and combinations on SCM scores in middle-aged mice (Experiment 1).** SCM scores are expressed as mean ± SE. SCM scores are obtained by calculation (% of correct responses – % of interferent responses). As can be seen, the lowest S 38093 dose combined with the highest Donepezil dose (DON 2 + S1), and inversely (DON 1 + S3), significantly increased SCM scores as compared to Vehicle and compounds alone. ^∗∗^*p* < 0.01 and ^∗∗∗^*p* < 0.001 as compared to vehicle; ^##^*p* < 0.05 and ^###^*p* < 0.01 respectively as compared to compounds alone.

*Strength of contextual memory (SCM)*. Data are presented in **Figure 3**. A one-way analysis of variance (ANOVA) showed a highly significant between-groups difference [*F*(11,132) = 9.76; *p* = 0.0001]. For compounds alone, S 38093 at 0.1 and 1.0 mg/kg and Donepezil at all doses did not show significant effect on the SCM scores as compared to the vehicle group (*p* > 0.10). For combinations of compounds, except the combination of the two lowest (Don at 0.1 mg/kg and S 38093 at 0.1 mg/kg) and the two highest doses (Don at 0.3 mg/kg and S 38093 at 1.0 mg/kg) of each compound, there was a strong, significant increase of SCM scores (*p* < 0.01, *p* < 0.001, *p* < 0.001, and *p* < 0.01, respectively).

#### Pharmacokinetics Interaction

Results have been summarized in **Table 1**. The inter-individual variability on S 38093 plasma concentrations was moderate. Cmax was reached 0.5 h after dosing and the elimination half-life was between 1.5 and 2.4 h. Both exposure and C_max_ increase in a dose proportional manner within the same treatment regimen.

**Table 1:** Mean pharmacokinetic parameters of S 38093 (0, 0.1, and 1.0 mg/kg) and Donepezil (0.3 mg/kg), given alone or in combination in middle-aged mice (five groups, *n* = 3/group).

	Dose	0	0.1		1	
S 38093	AUC (ng.h/mL)	–	9.56	3.69	92.40	53.90
	Cmax (ng/mL)	–	3.04	1.27	55.6	36.30
	Tl/2(h)	–		1.51	2.37	1.54
	Tmax(ll)	–	2.0	0.5	0.5	0.5

	Dose	0.3	0	0.3	0	0.3

Donepezi	AUC (ng.h/mL)	2.79	–	0.90	–	1.55
	Cmax (ng/mL)	0.96	–	0.48	–	1.11
	Tmax(ll)	1.0	–	0.5	–	0.5

*The PK parameters of the co-administration of S 38093 (0.1 and 1.0 mg/kg) with Donepezil (0.3 mg/kg) are in the gray columns.*

Variability of Donepezil plasma concentrations following concomitant administration of 0.3 mg/kg was moderate. Maximal Donepezil plasma concentrations were observed between 0.5 and 1 h after administration and were similar with and without co-administration of S 38093. There was no increase of neither S 38093 nor Donepezil plasma exposure when administered as a combination.

### Experiment 2: Study of the Effect of the Compounds on Age-Related Memory Deficit and CREB Phosphorylation

Body weights among the groups were ranged from 25.9 ± 2.8 g (young adult mice) to 31.6 ± 4.1 g and no significant between-groups difference was observed (*p* < 0.12). During the food restriction period, both vehicles and drug-treated mice eaten all the allocated daily amount of dry food. As in Experiment 1, all animals retained for the test session have eaten at least 7/8 pellets out of 10 at both the first and second acquisitions.

#### Effects of Aging on SCM Score and pCREB Activity

##### Behavior

*Acquisition phase*. No significant difference was observed among the groups on the total number of head-dips as well as on the % exploration of the baited hole both at acquisitions 1 and 2 [*F*(1,18) < 1.0 in all analyses; data not shown].

##### Test session

**FIGURE 4:**
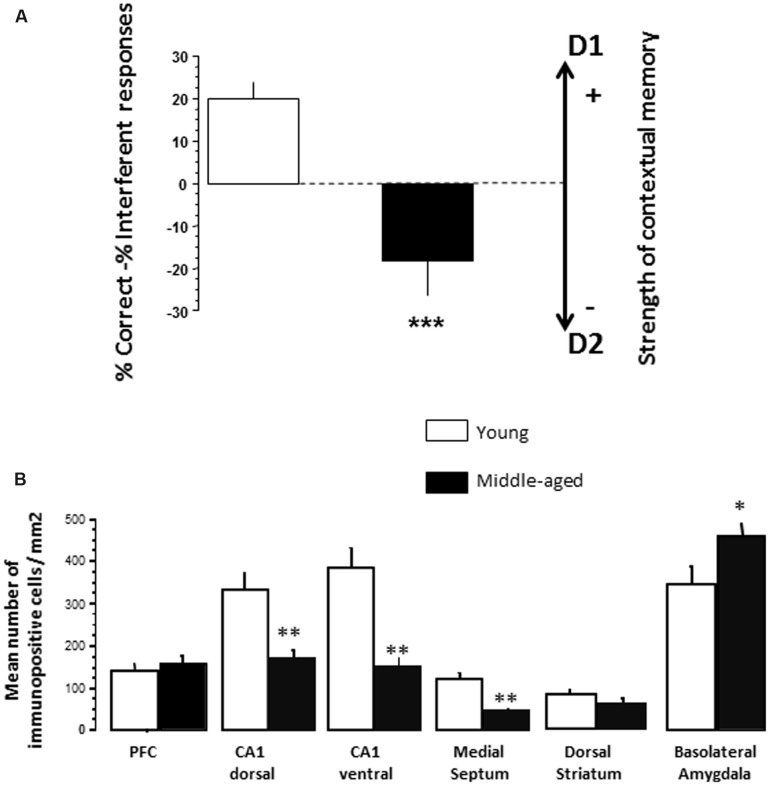
**(A)** Effects of aging on SCM scores (Experiment 2). As can be seen, 14 months-old middle-aged mice exhibited a significant reduction of SCM score as compared to 5 months-old young-adult mice. Between-groups difference: ^∗∗∗^*p* < 0.001. **(B)** Effects of aging on pCREB immunoreactivities. Counting was made in the PFC, dCA1, vCA1, medial septum, dorsal striatum, and BLA. No significant differences were observed between the groups in all brain areas in naïve condition (data not shown). Middle-aged mice showed reduced behavioral testing-related pCREB immunoreactivities in the dCA1, vCA1, and medial septum as compared to young adult mice. In contrast, a significant increase of pCREB was observed in the BLA in middle-aged animals, as compared to young ones. Results are expressed as mean number of positive pCREB nuclei per mm^2^. ^∗^*p* < 0.05, ^∗∗^*p* < 0.01.

*Effects of aging on SCM scores*. Results are presented in **Figure 4A**. Young adults and middle-aged animals exhibited a similar total number of head-dips at the test session [30 ± 4.26 and 25.5 ± 4.05, respectively; Groups: *F*(1,18) < 1.0]. However, young mice exhibited a positive score (+20.0 ± 3.6) whereas middle-aged animals exhibited a negative one (-18.9 ± 7.9) [Groups; *F*(1,18) = 18.92; *p* = 0.0004].

##### Immunohistochemistry

*Effects of aging on tCREB and pCREB immunoreactivity*. Results are presented in **Figure 4B**. One way ANOVA evidenced no significant difference (*p* > 0.10 in all analyses) between young adult and middle-aged mice on the number of tCREB immunopositive cells in naïve and test conditions, whatever the brain areas counted (data not shown). Concerning pCREB, one way ANOVA also evidenced no significant between-groups difference in naïve condition on pCREB scores whatever the brain areas counted (*p* > 0.10 in all analyses; data not shown).

#### Effects of Treatments on SCM Scores and pCREB Activity in Middle-Aged Mice

##### Behavior

The group of young adult mice has been discarded from further statistical analyses since the effects of the compounds alone or in combination have been studied only in middle-aged animals.

*Acquisition phase*. No significant difference was observed among the groups on the total number of head-dips as well as on the % exploration of the baited hole both at acquisitions 1 and 2 [*F*(1,54) < 1.0 in all analyses; data not shown].

##### Test phase

*Total number of head-dips*. A significant between-groups difference is observed [*F*(5,54) = 2.95; *p* = 0.02] being mainly due to the S 0.3 mg/kg (44.90 ± 6.56) and S 0.1 mg/kg (37.4 ± 6.15) groups. However, *post hoc* analyses failed to reach the statistical level of significance for these two groups as compared to either vehicles (25.50 ± 4.05; *p* > 0.10) or Don 0.3 mg/kg (23.90 ± 4.06; *p* > 0.15). The groups receiving the doses combinations (Don 0.3 mg/kg+ S 0.1 mg/kg: 21.90 ± 3.42; Don 0.3 mg/kg + S 0.3 mg/kg: 29.10 ± 6.06) exhibited a total number of head-dips similar to that of vehicles (*p* > 0.10).

**FIGURE 5:**
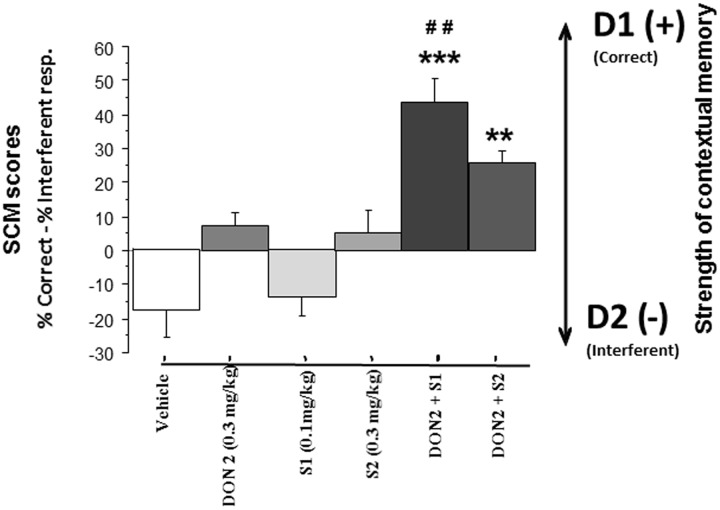
**Effects of S 38093, Donepezil and combinations on SCM scores in middle-aged mice (Experiment 2).** SCM scores are expressed as mean ± SE. SCM scores are obtained by calculation (% of correct responses – % of interferent responses). As can be seen, the lowest S 38093 dose combined with Donepezil significantly increased SCM scores as compared to Vehicle and compounds (Donepezil and S 38093) alone. Combination of Donepezil and the higher S 38093 dose induced a significant increase of SCM score as compared to vehicle only. ^∗∗^*p* < 0.01 and ^∗∗∗^*p* < 0.001 as compared to vehicle; ##*p* < 0.01 as compared to Donepezil and S 38093 alone.

*Strength of contextual memory*. Data are represented in **Figure 5**. ANOVA evidenced significant effects of Donepezil [*F*(1,54) = 48.88; *p* < 0.0001], of S 38093 [*F*(2,54) = 7.49; *p* = 0.0013] and of the interaction between Donepezil and S 38093 [*F*(2,54) = 5.57; *p* = 0.0063]. Donepezil (+7.48 ± 3.60) and S 38093 at the doses of 0.3 mg/kg (+5.19 ± 6.68) and 0.1 mg/kg (-14.3 ± 5.76) produced no significant modifications of the scores as compared to vehicle (-18.14 ± 7.9; NS in all comparisons). However, the higher positive scores were observed in groups receiving the combinations of Donepezil and S 38093 at the doses of 0.1 mg/kg (+43.59 ± 7.10; *p* < 0.001 versus vehicle and *p* < 0.01 versus the Donepezil group) and 0.3 mg/kg (+25.73 ± 3.96; *p* < 0.01 versus vehicle; NS versus Donepezil). Thus, only the combination of Donepezil and S 38093 at 0.1 mg/kg produced a significant increase of SCM score as compared to both vehicle-treated mice and Don 0.3 mg/kg.

In contrast, significant differences were observed in test condition (**Figure 5**). More specifically, middle-aged mice exhibited less immunopositive cells as compared to young animals in the dCA1 (-47.5%; *p* = 0.007), the vCA1 (-56%; *p* = 0.0037), and the MS (-66.2%; *p* = 0018). A weak increase of immunopositive cells is observed in the BLA of middle-aged mice (+32.7%; *p* = 0.039). No significant between-groups difference was observed in the PFC and the dorsal striatum.

##### Effects of treatments on tCREB and pCREB immunoreactivity in middle-aged mice

*Total CREB*. Analysis of variance analyses showed no significant between-groups difference, no treatments effect and no significant interaction between drugs were found both in naïve and test conditions (*p* > 0.10 in all analyses; data not shown).

*Phosphorylated CREB*. In naïve condition, no significant between-groups difference, no treatments effect and no significant interaction between drugs was found whatever the brain structure counted (*p* > 0.10 in all analyses; data not shown).

**FIGURE 6:**
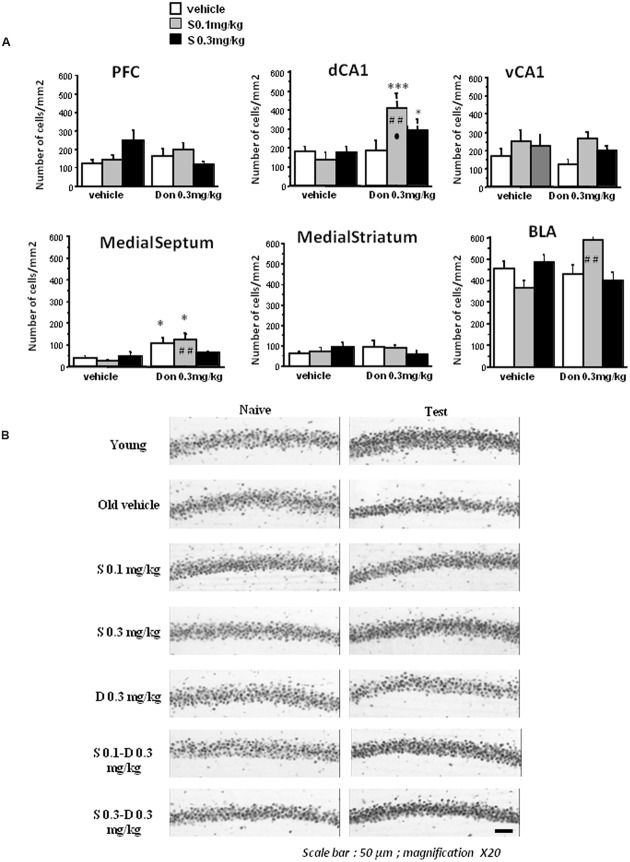
**Effects of S 38093, Donepezil and combinations on pCREB immunoreactivities in middle-aged mice. (A)** Counting was made in the PFC, dCA1, vCA1, medial septum, dorsal striatum, and BLA. No significant differences were observed between the groups in all brain areas in naïve condition (data not shown). As can be seen, the lowest S 38093 dose combined with Donepezil significantly increased pCREB immunopositive cells as compared to vehicles and compounds (Donepezil and S 38093) alone. A significant increase of immunopositive cells was also observed in the BLA with the same combination as compared to compounds alone (Donepezil or S 38093). Combination of Donepezil and the higher S 38093 dose induced a significant increase of pCREB immunopositive cells as compared to vehicles only. ^∗^*p* < 0.05 and ^∗∗∗^*p* < 0.001 as compared to vehicles; ##*p* < 0.01 as compared to Donepezil and ^∙^*p* < 0.05 as compared to S 38093 alone. **(B)** Representative photomicrographs showing pCREB immunoreactivities in the dCA1 of the hippocampus in young adult and middle-aged mice after administration of S 38093 (0.1 and 0.3 mg/kg), Donepezil (0.3 mg/kg) and combinations both in naïve and test conditions. Magnification X20, Scale bar: 50 μm.

In test condition (**Figure 6A**), no significant between-groups difference and no significant interaction between drugs were observed in the BLA, the dorsal striatum and the vCA1 (*p* > 0.10 in all analyses). However, donepezil induced a significant increase of immunopositive cells in the dCA1 [*F*(1,54) = 11.2; *p* < 0.001] and in the MS [*F*(1,54) = 18.3; *p* < 0.001]. S 38093 at both doses has no significant effect in these two brain areas. Two way ANOVA also showed that S38093 and Donepezil have a significant interaction effect in the dCA1 [*F*(2,54) = 3.57; *p* < 0.03] and the interaction is near from statistical significance in the MS [*F*(2,54) = 3.0; *p* = 0.058]. **Figure 6B** illustrated changes in pCREB activity in the dCA1 of the hippocampus in the different groups in naïve and test conditions.

Calculus in percentage of the enhancement of pCREB in the combination groups as compared to compounds alone or vehicles were performed. The main findings are as follows:

*In the BLA*, Dunnett *post hoc* test evidenced a significant increase of pCREB expression in mice receiving S 0.1 mg/kg +Don 0.3 mg/kg (+60.9%) in comparison with S 0.1 mg/kg alone (*p* < 0.01).

*In the dCA1*, results showed a significant increase of pCREB expression in mice receiving the combination S 0.1 mg/kg+ Don 0.3 mg/kg (+129.4%) and in mice receiving S 0.3 mg/kg+ Don0.3 mg/kg (+64.7%; *p* < 0.01) as compared to vehicle. More importantly, for the group receiving the combination S 0.1 mg/kg+Don0.3 mg/kg, a significant increase of pCREB expression of +192.7% has been evidenced as compared to S 0.1 mg/kg alone (*p* < 0.01) and of +137.1% in comparison with Donepezil alone (*p* < 0.01).

*In the MS*, Donepezil alone as well as the combination S 0.1 mg/kg+ Don 0.3 mg/kg induced a significant increase of pCREB expression of +174.3% and +214.1% respectively as compared to vehicle. A significant increase of +380.7% has also been evidenced between S 0.1 mg/kg alone and the combination S 0.1+ Don 0.3 mg/kg.

## Discussion

### Synergistic Effects between Donepezil and S 38093 on Contextual Memory in Middle-Aged Mice

As regards SCM scores, vehicle-treated middle-aged mice showed a negative score on the retention of D1 (indicating an increase of interferent responses) in sharp contrast to young adult mice which exhibited a very positive score. In contrast, compounds alone or in combination (excepted for the combinations of the two lowest and the two highest doses of each compound) reverse this age-related memory pattern, as those mice have a substantial memory of D1 but not of D2 as compared to vehicle-treated animals. Since it has been shown that memory of D1 is hippocampus-dependent (Chauveau et al., 2008, 2010) whereas memory of D2 is dependent on the PFC activity (Chauveau et al., 2009), it is thus of importance to verify that any promnesiant impact of the combinations of compounds on D1 did not alter memory of D2. This effect, statistically significant for the combinations with the exception quoted above, is the index of a substantial contextual memory-enhancing effect since the increase of the correct response is accompanied by a concomitant decrease of the interference one. Interestingly, the more powerful combination is observed with the lowest S 38093 dose + Donepezil 0.3 mg/kg since this combination induced a significant enhancement of contextual memory as regards to both Donepezil and S 38093 alone. It is noteworthy that combination of S 38093 with low pro-cognitive doses of any other compound currently approved for moderate-to-severe AD [i.e., others AChEI (rivastigmine and galantamine) and memantine, an uncompetitive NMDA (*N*-methyl-D-aspartate) antagonist] also leads to higher reversal of age-related memory deficit in this task compared to compounds alone (unpublished results, data not shown). Moreover, there was a very good correlation between the increase of percentage of correct responses and the decrease of percentage of interferent responses, confirming the specific effect of each compound and their combinations on contextual memory (data not shown).

Interestingly, a synergistic effect between a H3 antagonist and an anti-cholinesterase inhibitor has also been described in cognitive impairment associated with scopolamine in healthy young subjects (Cho et al., 2011). Our data showing synergistic effects between the H3 antagonist S 38093 and drugs approved in AD are innovative since, to our knowledge, that has never been described for other H3 compounds nor procognitive compounds on natural model of age-induced amnesia in rodents.

As the two compounds were administered simultaneously, it was important to assess a potential pharmacokinetic interaction, which could have faked a synergistic effect by increasing the blood exposure. Our study confirmed that the synergistic effect of the combination of the two compounds is not due to a pharmacokinetic interaction between them. Moreover, the safety of S 38093 and Donepezil, alone or in combination, was tested using the primary observation (IRWIN) test in mice and results showed that the combination of pharmacological doses of both compounds did not induce any observable clinical signs, similarly as compounds administered alone (data not shown).

### Synergistic Effects between Donepezil and S 38093 on pCREB Immunoreactivity in the Dorsal Hippocampus and the Medial Septum

Aging reduced pCREB in the dCA1, the vCA1 and the MS as compared to young vehicle, whereas an increase of pCREB is observed in the BLA. These data confirm that alterations of the hippocampus activity induced contextual memory deficit in middle-aged animals (Béracochéa et al., 2011). Interestingly, we also previously showed that the BLA and the mPFC are importantly involved in the memory retrieval of D2 (Chauveau et al., 2009; Dominguez et al., 2014). Thus, the increase of pCREB in the BLA during memory retrieval of D1 could reflect an abnormal concomitant recruitment of a BLA-PFC network in middle-aged animals which could increase interference (D2) responses.

Whereas no significant between-groups difference is observed in the naive condition, S 38093 and Donepezil have a significant synergistic effect in the dCA1 and near significant effect in the MS, as compared to each compound alone. More precisely, the combination of S 0.1 mg/kg and Donepezil 0.3 mg/kg reverses in the dCA1 the age-induced hypo-phosphorylation of pCREB. This combination is also the most efficacious in reversing the age-induced memory retrieval deficit for D1, as previously reported.

### Hypothesis on the Mechanism of Action

Immunohistochemical data show that the combination of S 0.1 mg/kg and Don 0.3 mg/kg increases pCREB in structures of the cholinergic septo-hippocampal loop. Indeed, the medial septal area provides most of the cholinergic innervation of the hippocampus (Jakab and Septum, 1995; Mamad et al., 2015).

Thus, one hypothesis regarding the mechanisms of action of the compounds is an effect on the hippocampal cholinergic system. Indeed, in separate experiments, we showed that the acute as well as chronic administration of S 38093 in rats, by antagonizing presynaptic H_3_ receptors, has been shown to rapidly and dose-dependently increase the release of ACh in the ventral hippocampus and the PFC of rats (intracerebral microdialysis, see supplemental data and Panayi et al., 2014). On the other hand, the well-known AChEI Donepezil, given alone, also increased ACh level in the synaptic cleft after acute administration. Previous studies have also confirmed that the increase in ACh levels induced by Donepezil in the cortex and hippocampus of rats are maintained after chronic administration (Scali et al., 2002). Either action could result in the observed enhancement of contextual memory with compounds alone, whereas the stronger effect of the combinations could be attributed to a synergistic effect on the hippocampal cholinergic system, including the additive effect on ACh release of the compounds observed in microdialysis when S 38093 and Donepezil were administered together (Supplemental Data, see Figures 1 and 2). Thus the microdialysis experiment evidenced the potential capacity of S 38093 and Donepezil to enhance the cholinergic activity within the hippocampo-PFC network that is substantially implicated in the CSD task.

Even if the effect of the compounds were evaluated in the PFC and the ventral hippocampus in supplemental data, it was also demonstrated that H3 antagonists including S 38093 (data no shown) as well as Donepezil were also able to significantly increase ACh levels in other brain areas and in particular dorsal hippocampus (Medhurst et al., 2007; Herrik et al., 2016), a critical region for memory processes, in which we have demonstrated pCREB increases in the present study.

Mice treated with the combination of the two highest doses of S 38093 and Donepezil showed impairments in the retrieval of D1. It could be hypothesized that this combination induces an important increase of acetylcholine levels both in the hippocampus and PFC, which could alter the interaction between these two areas during the testing of D1, leading to memory impairment. Indeed, the concomitant recruitment of the PFC by acetylcholine in mice receiving the drug combinations could enhance interference (D2) responses (Chauveau et al., 2009), at the expense of the dHPC-dependent one (D1).

## Conclusion

The present study shows that the combination of the two memory-enhancing compounds, Donepezil and the H_3_ antagonist S 38093, can lead to synergistic memory-enhancing effects, with a statistically higher size of effect never obtained with any memory-enhancing compounds alone without any pharmacokinetic interaction between both compounds. The memory-enhancing effect of the S 38093 and Donepezil combination is mediated by its action on the septo-hippocampal circuitry, since it canceled out the hypo-phosphorylation of pCREB in both the dCA1 and the medial septum that is observed in vehicle-treated middle-aged mice. Given the known pro-cholinergic effects of histaminergic H3 inverse agonists and Donepezil (Schwartz and Lecomte, 2016) and our own data drawn from the microdialysis experiment (supplemental data), it could be suggested that the synergistic effects of the combination of S 38093 and Donepezil on memory performance in middle-aged mice could stem from an enhancement of the septo-hippocampal cholinergic system.

## Author Contributions

DB: planning pharmacological and immunohistochemical experiments and writing of the manuscript; AS: planning pharmacological experiments and writing of the manuscript; JK: planning biochemical experiments; TY: planning and running microdialysis experiments; GD: running immunohistochemical experiments; ND: running immunohistochemical experiments; CL: planning of experiments and supervising the study; EM: supervisor and writing of the manuscript; AK: running pharmacological experiments.

## Conflict of Interest Statement

These studies were financially supported by Servier (Suresnes, France). AS and EM are currently employees of Servier. All the other authors declare that the research was conducted in the absence of any commercial or financial relationships that could be construed as a potential conflict of interest.

## References

[B1] Arrang J. M. Garbarg M. Schwartz J. C. (1983). Auto-inhibition of brain histamine release mediated by a novel class (H3) of histamine receptor. *Nature* 302 832–837. 10.1038/302832a0 6188956

[B2] Barsegyan A. Mackenzie S. M. Kurose B. D. McGaugh J. L. Roozendaal B. (2010). Glucocorticoids in the prefrontal cortex enhance memory consolidation and impair working memory by a common neural mechanism. *Proc. Natl. Acad. Sci. U.S.A.* 107 16655–16660. 10.1073/pnas.1011975107 20810923 PMC2944727

[B3] Bartus R. T. Dean R. L. III Beer B. Lippa A. S. (1982). The cholinergic hypothesis of geriatric memory dysfunction. *Science* 217 408–414. 10.1126/science.7046051 7046051

[B4] Baudonnat M. Guillou J. L. Husson M. Vandesquille M. Corio M. Decorte L. (2011). Disrupting effect of drug-induced reward on spatial but not cue-guided learning: implication of the striatal protein kinase A/cAMP response element-binding protein pathway. *J. Neurosci.* 31 16517–16528. 10.1523/JNEUROSCI.1787-11.2011 22090478 PMC6633299

[B5] Benito E. Barco A. (2010). CREB’s control of intrinsic and synaptic plasticity: implications for CREB-dependent memory models. *Trends Neurosci.* 33 230–240. 10.1016/j.tins.2010.02.001 20223527

[B6] Béracochéa D. Philippin J. N. Meunier S. Morain P. Bernard K. (2007). Improvement of episodic contextual memory by S 18986 in middle-aged mice: comparison with donepezil. *Psychopharmacology* 193 63–73. 10.1007/s00213-007-0765-4 17384936

[B7] Béracochéa D. Philippin J. N. Meunier S. Morain P. Bernard K. (2008). Improvement of episodic contextual memory by S 18986 in middle-aged mice: comparison with Donepezil. *Psychopharmacology* 196 555–564. 10.1007/s00213-007-0987-5 17384936

[B8] Béracochéa D. Tronche C. Coutan M. Dorey R. Chauveau F. Piérard C. (2011). Interaction between Diazepam and hippocampal corticosterone after acute stress: impact on memory in middle-aged mice. *Front. Behav. Neurosci.* 12:14 10.3389/fnbeh.2011.00014 PMC3079857 21516247

[B9] Bernabeu R. Bevilaqua L. Ardenghi P. Bromberg E. Schmitz P. Bianchin M. (1997). Involvement of hippocampal cAMP/cAMPdependent protein kinase signaling pathways in a late memory consolidation phase of aversively motivated learning in rats. *Proc. Natl. Acad. Sci. U.S.A.* 94 7041–7046. 10.1073/pnas.94.13.7041 9192688 PMC21281

[B10] Blandina P. Giorgetti M. Bartolini L. Cecchi M. Timmerman H. Leurs R. (1996). Inhibition of cortical acetylcholine release and cognitive performance by histamine H3 receptor activation in rats. *Br. J. Pharmacol.* 119 1656–1664. 10.1111/j.1476-5381.1996.tb16086.x 8982515 PMC1915786

[B11] Brown R. E. Stevens D. R. Haas H. L. (2001). The physiology of brain histamine. *Prog. Neurobiol.* 63 637–672. 10.1016/S0301-0082(00)00039-3 11164999

[B12] Chauveau F. Piérard C. Coutan M. Drouet I. Liscia P. Béracochéa D. (2008). Prefrontal cortex or basolateral amygdala lesions blocked the stress-induced inversion of serial memory retrieval pattern in mice. *Neurobiol. Learn. Mem.* 90 395–403. 10.1016/j.nlm.2008.04.014 18572424

[B13] Chauveau F. Pierard C. Tronche C. Coutan M. Drouet I. Liscia P. (2009). The hippocampus and prefrontal cortex are differentially involved in serial memory retrieval in non-stress and stress conditions. *Neurobiol. Learn. Mem.* 91 447–455. 10.1016/j.nlm.2008.12.003 19110063

[B14] Chauveau F. Tronche C. Piérard C. Liscia P. Drouet I. Coutan M. (2010). Rapid stress-induced corticosterone rise in the hippocampus reverses serial memory retrieval pattern. *Hippocampus* 20 196–207. 10.1002/hipo.20605 19360856

[B15] Cho W. Maruff P. Connell J. Gargano C. Calder N. Doran S. (2011). Additive effects of a cholinesterase inhibitor and a histamine inverse agonist on scopolamine deficits in humans. *Psychopharmacology (Berl.).* 218 513–524. 10.1007/s00213-011-2344-y 21644059

[B16] Colombo P. J. Brightwell J. J. Countryman R. A. (2003). Cognitive strategy-specific increases in phosphorylated cAMP response element-binding protein and c-Fos in the hippocampus and dorsal striatum. *J. Neurosci.* 23 3547–3554. 12716964 10.1523/JNEUROSCI.23-08-03547.2003 PMC6742292

[B17] Dominguez G. Dagnas M. Decorte L. Vandesquille M. Belzung C. Béracochéa D. (2016). Rescuing prefrontal camp-creb pathway reverses working memory deficits during withdrawal from prolonged alcohol exposure. *Brain Struct. Funct.* 221 865–877. 10.1007/s00429-014-0941-3 25388276

[B18] Dominguez G. Faucher P. Henkous N. Krazem A. Piérard C. Béracochéa D. (2014). Stress induced a shift from dorsal hippocampus to prefrontal cortex dependent memory retrieval: role of regional corticosterone. *Front. Behav. Neurosci.* 8:166 10.3389/fnbeh.2014.00166 PMC4030165 24860451

[B19] Femenía T. Magara S. DuPont C. M. Lindskog M. (2015). Hippocampal-dependent antidepressant action of the H3 receptor antagonist clobenpropit in a rat model of depression. *Int. J. Neuropsychopharmacol.* 18 yv032 10.1093/ijnp/pyv032 PMC4576519 25762718

[B20] Fernández-Novoa L. Cacabelos R. (2001). Histamine function in brain disorders. *Behav. Brain Res.* 124 213–233. 10.1016/S0166-4328(01)00215-7 11640975

[B21] Fox G. B. Pan J. B. Radek R. J. Lewis A. M. Bitner R. S. Esbenshade T. A. (2003). Two novel and selective nonimidazole H3 receptor antagonists A-304121 and A-317920: II. In vivo behavioral and neurophysiological characterization. *J. Pharmacol. Exp. Ther.* 305 897–908. 12606600 10.1124/jpet.102.047241

[B22] Francis P. T. Ramírez M. J. Lai M. K. (2010). Neurochemical basis for symptomatic treatment of Alzheimer’s disease. *Neuropharmacology* 59 221–229. 10.1016/j.neuropharm.2010.02.010 20156462

[B23] Fu A. Zhou R. Xu X. (2014). The synthetic thyroid hormone, levothyroxine, protects cholinergic neurons in the hippocampus of naturally aged mice. *Neural Regen. Res.* 15 864–871. 10.4103/1673-5374.131602 PMC4146262 25206902

[B24] Herrik K. F. Mørk A. Richard N. Bundgaard C. Bastlund J. F. de Jong I. E. (2016). The 5-HT6 receptor antagonist idalopirdine potentiates the effects of acetylcholinesterase inhibition on neuronal network oscillations and extracellular acetylcholine levels in the rat dorsal hippocampus. *Neuropharmacology* 107 351–363. 10.1016/j.neuropharm.2016.03.043 27039041

[B25] Jakab R. L. Septum L. C. (1995). “The hippocampal formation,” in *The Rat Nervous System*, 2nd Edn, ed. Paxinos G. (Cambridge, MA: Academic Press), 405–442.

[B26] Johannsson M. Snaedal J. Johannesson G. H. Gudmundsson T. E. Johnsen K. (2015). The acetylcholine index: an electroencephalographic marker of cholinergic activity in the living human brain applied to Alzheimer’s disease and other dementias. *Dement. Geriatr. Cogn. Disord.* 39 132–142. 10.1159/000367889 25471612

[B27] Ligneau X. Landais L. Perrin D. Piriou J. Uguen M. Denis E. (2007). Brain histamine and schizophrenia: potential therapeutic applications of H3-receptor inverse agonists studied with BF2.*649*. *Biochem. Pharmacol.* 73 1215–1224. 10.1016/j.bcp.2007.01.023 17343831

[B28] Lim Y. Y. Maruff P. Schindler R. Ott B. R. Salloway S. Yoo D. C. (2015). Disruption of cholinergic neurotransmission exacerbates Aβ-related cognitive impairment in preclinical Alzheimer’s disease. *Neurobiol. Aging* 36 2709–2715. 10.1016/j.neurobiolaging.2015.07.009 26233262

[B29] Lockhart B. P. Lestage P. J. (2003). Cognition enhancing or neuroprotective compounds for the treatment of cognitive disorders: why? when? which? *Exp. Gerontol.* 38 119–128. 10.1016/S0531-5565(02)00163-8 12543269

[B30] Mamad O. McNamara H. M. Reilly R. B. Tsanov M. (2015). Medial septum regulates the hippocampal spatial representation. *Front. Behav. Neurosci.* 30:166 10.3389/fnbeh.2015.00166 PMC4485312 26175674

[B31] Marighetto A. Valerio S. Desmedt A. Philippin J. N. Trocmé-Thibierge C. Morain P. (2008). Comparative effects of the alpha7 nicotinic partial agonist, S 24795, and the cholinesterase inhibitor, Donepezil, against aging-related deficits in declarative and working memory in mice. *Psychopharmacology (Berl.).* 197 499–508. 10.1007/s00213-007-1063-x 18265960

[B41] Medhurst A. D. Atkins A. R. Beresford I. J. Brackenborough K. Briggs M. A. Calver A. R. (2007). GSK189254, a novel H_3_ receptor antagonist that binds to histamine H_3_ receptors in Alzheimer’s disease brain and improves cognitive performance in preclinical models. *J. Pharmacol. Exp. Ther.* 321 1032–1045. 10.1124/jpet.107.120311 17327487

[B32] Panayi F. Sors A. Bert L. Martin B. Rollin-Jego G. Billiras R. (2014). “In vivo pharmacological profile of S 38093, a novel inverse agonist at histamine H3 receptors,” *in Poster at the Neuroscience Meeting Planner: Program N° 21.08SA/UU28*. (Washington, DC: Society for Neuroscience).

[B33] Paxinos G. Franklin K. B. J. (2001). *The Mouse Brain in Stereotaxic Coordinates*, 2nd Edn. Cambridge, MA: Academic Press.

[B34] Runyan J. D. Dash P. K. (2005). Distinct prefrontal molecular mechanisms for information storage lasting seconds versus minutes. *Learn. Mem.* 12 232–238. 10.1101/lm.92405 15930501 PMC1142450

[B35] Scali C. Casamenti F. Bellucci A. Costagli C. Schmidt B. Pepeu G. (2002). Effect of subchronic administration of metrifonate, rivastigmine and donepezil on brain acetylcholine in aged F344 rats. *J. Neural. Transm. (Vienna).* 109 1067–1080. 10.1007/s007020200090 12111444

[B36] Schwartz J. C. (2011). The histamine H3 receptor: from discovery to clinical trials with pitolisant. *Br. J. Pharmacol.* 163 713–721. 10.1111/j.1476-5381.2011.01286.x 21615387 PMC3111674

[B37] Schwartz J. C. Lecomte J. M. (2016). Clinical trials with H3-receptor inverse agonists: what they tell us about the role of histamine in the human brain. *Neuropharmacology* 106 35–36. 10.1016/j.neuropharm.2016.04.006 27060410

[B38] Tronche C. Lestage P. Louis C. Carrie I. Béracochéa D. (2010). Pharmacological modulation of contextual “episodic-like” memory in aged mice. *Behav. Brain Res.* 215 255–260. 10.1016/j.bbr.2010.04.009 20385172

[B39] Vandesquille M. Baudonnat M. Decorte L. Louis C. Lestage P. Béracochéa D. (2013). Working memory deficits and related disinhibition of the cAMP/PKA/CREB are alleviated by prefrontal α4β2^∗^-nAChRs stimulation in aged mice. *Neurobiol. Aging* 34 1599–1609. 10.1016/j.neurobiolaging.2012.10.006 23352115

[B40] Witkin J. M. Nelson D. L. (2004). Selective histamine H3 receptor antagonists for treatment of cognitive deficiencies and other disorders of the central nervous system. *Pharmacol. Ther.* 103 1–20. 10.1016/j.pharmthera.2004.05.001 15251226

