# Supplementary material for: The Synergistic Enhancing-Memory Effect of Donepezil and S 38093 (a Histamine H3 Antagonist) Is Mediated by Increased Neural Activity in the Septo-hippocampal Circuitry in Middle-Aged Mice
Source: Front Pharmacol. 2016 Dec 22;7:492. doi: 10.3389/fphar.2016.00492 (PMC5177663; doi:10.3389/fphar.2016.00492)
Supplement: Supplementary file 1 [file Data_Sheet_1.docx]

**Supplemental data**

**Intracerebral microdialysis: effects of S 38093 on acetylcholine concentrations in the hippocampus and prefrontal cortex in rats**.

The effects of acute administration of S 38093, alone or in combination with Donepezil, on acetylcholine extracellular levels were analyzed in the ventral hippocampus and medial prefrontal cortex of freely moving rats by microdialysis to investigate possible synergistic effects. The locomotor activity of the rats was recorded simultaneously during the sampling as a control method to assess a possible contribution of the handling stress and drug administration to microdialysis data.

**Material and methods. Surgery and microdialysis experiments**

Male Wistar (Han) rats, weighting about 265 g, were used for the experiment. At their arrival at facility, rats were housed in humidity and temperature controlled room on a 12h light/dark cycle (light on at 6:00 h) with water and food available ad libitum. Rats were anaesthetized with isoflurane and stereotaxically implanted with two guide cannula: one into the ventral hippocampus (vHPC: Interaural + 3.7 mm, Lateral + 4.8 mm, Ventral -3.8 mm providing the final -8.0 mm for the tip of the microdialysis probe) and the second into the medial prefrontal cortex (mPFC: Interaural + 12.2 mm, Lateral - 0.5 mm, Ventral -1.6 mm providing the final -5.6 mm for the tip of the microdialysis probe). After a recovery of 5 days, a microdialysis probe was inserted into the each respective guide cannula of awake rat. The probes were perfused at a constant flow-rate of 1 µl/min with sterile artificial CSF solution in the absence of an AChE inhibitor (148 mM NaCl, 4 mM KCl, 0.8 mM MgCl2, 1.4 CaCl2, 1.2 mM Na2HPO4, 0.3 mM NaH2PO4, pH 7.2). Following 120-min stabilization period, the samples were collected every 30 minutes. The first 3 samples (-90 to 0 min) were taken for determination of basal extracellular levels of ACh. Thereafter, S 38093 (3, 10 mg/kg) and Donepezil (0.1, 0.3 mg/kg), alone or in combination, or vehicle (saline) (9 groups, n=8/group) were administered i.p. and the samples were collected for the additional 3 hrs. After finalizing the experiment, the animals were sacrificed by an overdose of isoflurane and dislocation of the neck.

The levels of ACh in the microdialysates were determined by high-performance liquid chromatography (HPLC) linked to a postcolumn immobilized enzyme reactor followed by electrochemical detection. Briefly, the HPLC system (HTEC-500, Eicom Corp.) included a pulse-free microflow pump, a degasser and an amperometric detector equipped with a platinum electrode. The potential of the working electrode vs. Ag/AgCl ref. electrode was set to +0.45 V vs. an Ag/AgCl reference elctrode. Samples were injected by use of a CMA/200 Refrigerated Microsampler (CMA/Microdialysis). The chromatograms were recorded and integrated by use of a computerized data acquisition system (DataApex, Prague, Czech Republic). ACh was separated on a 150 x 2.0 I.D. mm narrow-bore column, packed with C18 polymer gel, 4 μm particle size. The enzyme reactor AC-ENZYMPAK II (Eicom Corp.) was used. The mobile phase was a 50 mM potassium hydrogen carbonate buffer containing 3.7 mM sodium 1-octanesulfonate, 0.13 mM EDTA-2Na, final pH 7.2. The detection limit (signal-to-noise ratio = 3) for ACh was 3 fmol in 15 µl injected onto the column. Determination of ACh levels in the groups of rats treated with drugs and vehicle was performed in blind conditions.

**Locomotor activity test**

Locomotor activity was monitored by use of a single-beam activity frame (44 x 30 cm ACTIMO 10, Shintechno, Japan) placed around the lower part of the Macrolon III cage. This arrangement allows for simultaneous recordings of locomotor activity and microdialysis sampling. The data were collected by counting and summarizing the overall activity (number of beam crossings) in 5-min intervals and further pooled into 30-min bins, thereby matching the frequency of microdialysis sampling.

### Statistical analysis

For each parameter of interest, descriptive statistics was performed on AUC baseline values (AUC_(-60-0 min)_ for mPFC and vHPC and AUC_(-120-0 min)_ for locomotor activity. The values on median, 1^st^ and 3^rd^ quartiles, N per group were calculated.

Differences between the groups treated with S 38093 + Donepezil compared to all other treated groups and a vehicle group in the mPFC and vHPC, respectively, were analyzed by two-way repeated measures (RM) ANOVA followed by Bonferroni’s multiple comparison test. Based on this analysis, the groups were divided into eight subgroups according to the doses of S 38093 + Donepezil combinations in the mPFC and vHPC, each subgroup included the (same) vehicle group. Statistical analysis (two-way repeated RM ANOVA followed by Bonferroni’s multiple comparison test) was carried-out.

**Results**

S 38093 (3 and 10 mg/kg i.p.) and Donepezil (0.1 and 0.3 mg/kg i.p.) given alone dose-dependently increased the ACh levels both in the mPFC and vHPC. These increases were rapid (from 30 min) and all significant except for S 38093 at 3 mg/kg in mPFC. The maximal increases induced by S 38093 (3 and 10 mg/kg i.p.) were reached at 30 min: 158% (n.s.) and 234% (p<0.001) of predrug values in mPFC, respectively, and 153% (p<0.001) and 184% (p<0.001) of predrug values in vHPC, respectively **(see Fig. 1 sup.data).**

The maximal increases induced by Donepezil (0.1 and 0.3 mg/kg i.p.) were reached at 30 min: 175% (p<0.01 ) and 216% (p<0.001) of predrug values in mPFC, respectively, and 180% (p<0.001) and 207% (p<0.001) of predrug values in vHPC, respectively **(see Fig. 1 sup.data).**

The combination of S 38093 at the dose of 10 mg/kg i.p, but not 3 mg/kg, and Donepezil at the dose of 0.1 mg/kg i.p., markedly potentiated the increases in extracellular ACh levels both in the mPFC and vHPC of awake rats when compared to the effects of any of the drugs given alone (at 30-60 min for Donepezil and 60 min for S 38093): maximum of 256% and 223% of the pre-drug values at 30 min in PFC and vHPC, respectively (p<0.001 and p<0.01) **(see Fig. 1 sup.data) (see Fig. 2 sup.data).**

The combination of S 38093 at 10 mg/kg i.p. but not 3 mg/kg, and Donepezil at 0.3 mg/kg i.p., markedly potentiated the increases in extracellular ACh levels both in the mPFC and vHPC of awake rats when compared to the effects of any of the drugs given alone: maximum of 344% and 307% of the pre-drug values at 30 min in PFC and vHPC, respectively (p<0.001) **(see Fig. 1 sup.data) (see Fig. 2 sup.data).**

Furthermore, it was observed that S 38093, Donepezil and the combined treatment of S 38093 + Donepezil significantly increased the locomotor activity at some points within the first 5-30 min as compared to the vehicle-treated group, however, there were no significant differences between the groups for the treatment and interaction between time and treatment (data not shown).
