# Supplementary figures and images for: The Synergistic Enhancing-Memory Effect of Donepezil and S 38093 (a Histamine H3 Antagonist) Is Mediated by Increased Neural Activity in the Septo-hippocampal Circuitry in Middle-Aged Mice
Source: Front Pharmacol. 2016 Dec 22;7:492. doi: 10.3389/fphar.2016.00492 (PMC5177663; doi:10.3389/fphar.2016.00492)

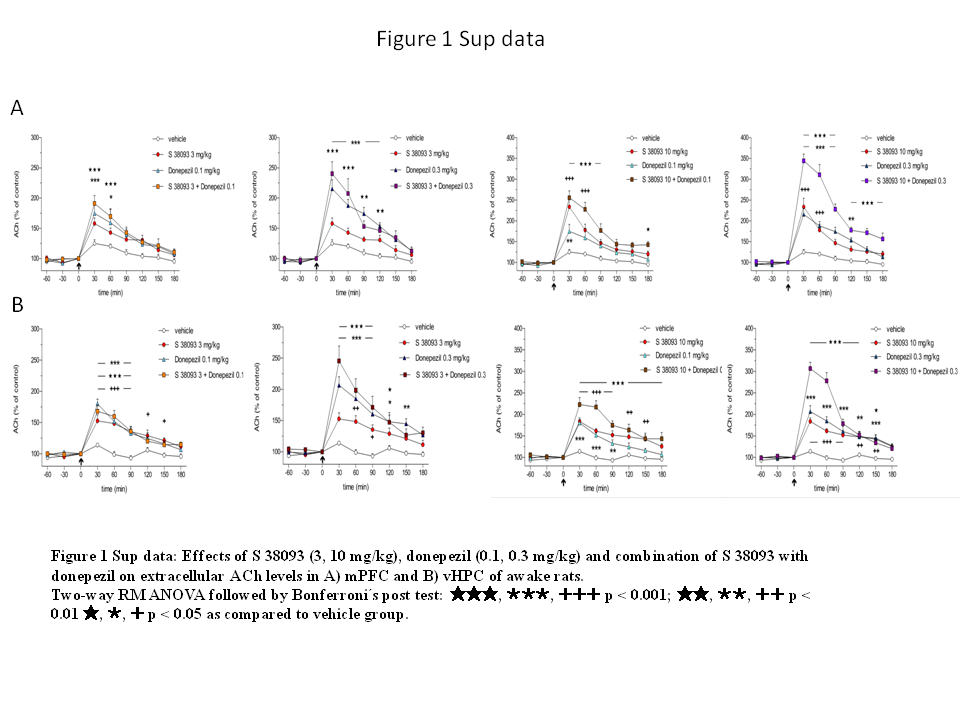

Supplement: Supplementary file 2 [file Image_1.TIF]

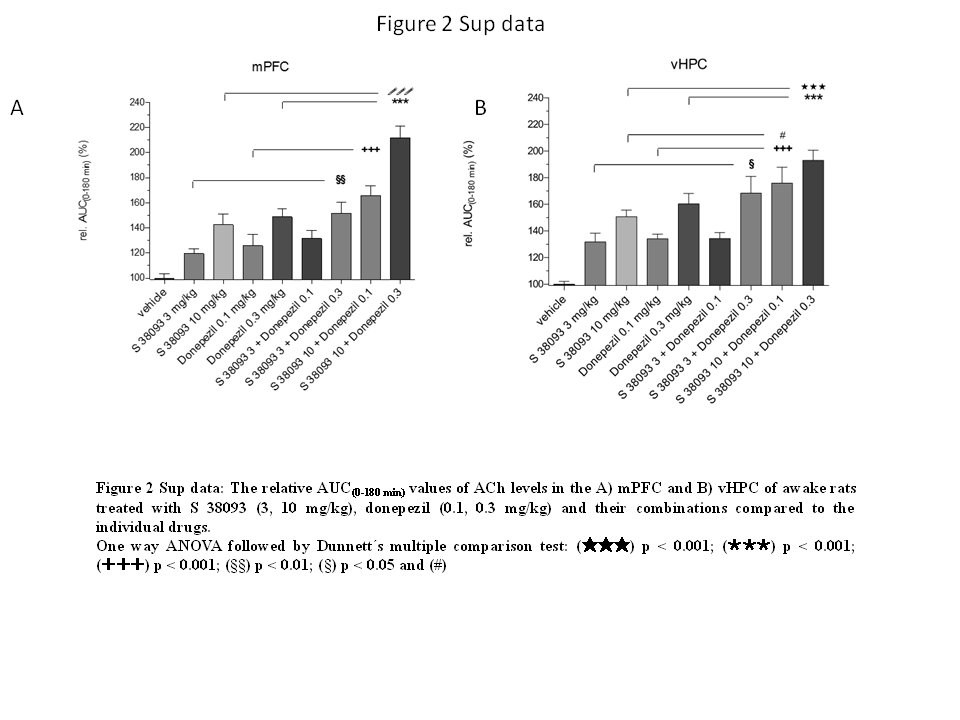

Supplement: Supplementary file 3 [file Image_2.TIF]
